# Association between the TNF-α-308G/A and TNF-α-238G/A polymorphisms and systemic lupus erythematosus susceptibility: an updated meta-analysis

**DOI:** 10.3389/fmed.2026.1836955

**Published:** 2026-06-23

**Authors:** Jiayi Wang, Yiwei Zhang, Lanfang Wang, Peng Huang

**Affiliations:** 1 Center for Evidence-Based Medicine, Jiangxi Provincial Key Laboratory of Disease Prevention and Public Health, School of Public Health, Jiangxi Medical College, Nanchang University, Nanchang, Jiangxi, China; 2 Queen Mary College, Jiangxi Medical College, Nanchang University, Nanchang, Jiangxi, China; 3 Electrocardiogram Diagnosis Room, The Second Affiliated Hospital, Jiangxi Medical College, Nanchang University, Nanchang, Jiangxi, China

**Keywords:** meta-analysis, polymorphism, susceptibility, systemic lupus erythematosus, TNF-α

## Abstract

**Objective:**

To perform an updated meta-analysis investigating the associations of TNF-α-308G/A and TNF-α-238G/A polymorphisms with systemic lupus erythematosus (SLE) susceptibility, incorporating the Bayesian false discovery probability (BFDP) and 95% prediction interval (PI) assessment to control for false-positive results.

**Methods:**

Case–control or cohort studies were identified through database searches up to October 16, 2025. Pooled odds ratios (ORs) with 95% confidence intervals (CIs) were calculated under five genetic models. Heterogeneity was assessed, and the BFDP and 95% PI was applied to evaluate significant associations.

**Results:**

Fifty-three studies were included concerning the TNF-α-308G/A polymorphism and 18 studies were selected on the TNF-α-238G/A polymorphism with SLE susceptibility. Overall, the TNF-α-308G/A polymorphism was associated with increased SLE susceptibility in overall populations, Asians, Caucasians, Indians, and mixed populations. No significant association was found for the TNF-α-238G/A polymorphism with SLE susceptibility. However, in the further sensitivity analysis, we observed that the TNF-α-238G/A polymorphism was associated with increased SLE susceptibility in overall analysis and mixed populations. Unfortunately, the 95% PI excluded the null value for only two of these significant association. However, BFDP values of four of these significant association were more than 0.80. Therefore, all significant associations were considered as less-credible positives in this study.

**Conclusion:**

This study indicates that the TNF-α-308G/A and TNF-α-238G/A polymorphisms may be not associated with increased SLE susceptibility. The statistically significant association with susceptibility to SLE is most likely due to false-positive results.

## 1 Introduction

Systemic lupus erythematosus (SLE) is a multifactorial autoimmune disorder characterized by chronic inflammation in various organs, with genetic factors influencing susceptibility (1–3). Twin and family studies indicate that genetic factors contribute to susceptibility to systemic lupus erythematosus (SLE) (4–6). Furthermore, numerous genes that are likely implicated in the development of SLE have been identified. Specific major histocompatibility complex (MHC) alleles, particularly HLA-A1, B8, and DR3, have been associated with SLE (7–9).

Tumor necrosis factor-alpha (TNF-α) is a pro-inflammatory cytokine produced by various cells, including macrophages, B cells, T cells, and mast cells (10). The role of TNF-α and its mechanisms of action in SLE remain incompletely elucidated. Nonetheless, TNF-α exhibited a significant correlation with various inflammatory and autoimmune diseases, which are the predominant disorders linked to the susceptibility of systemic lupus erythematosus (SLE) (11). Moreover, accumulating evidence suggests that TNF-α plays a dual role in immune regulation. On one hand, TNF-α promotes inflammatory responses by activating specific signaling pathways, contributing to tissue damage in target organs such as the kidney and skin. On the other hand, some studies have proposed that TNF-α may exert immunomodulatory effects by regulating T-cell apoptosis and dendritic cell maturation, potentially influencing disease activity in a context-dependent manner (12, 13). This functional duality underscores the importance of genetic variants that modulate TNF-α expression in determining SLE susceptibility. The single-nucleotide polymorphisms TNF-α-308G/A (rs1800629) and TNF-α-238G/A (rs361525) are situated on chromosome 6p21.3, within the class III region of the major histocompatibility complex (MHC), and directly influence gene regulation, having been linked to modified transcriptional activity of TNF-α in numerous disorders (14, 15).

Fifty-two articles and seven previously published meta-analyses have been conducted to examine the relationship between TNF-α-308G/A and TNF-α-238G/A polymorphisms and the susceptibility of SLE. Nonetheless, their results remain inconsistent. Notably, numerous original studies were not included in seven previously published meta-analyses, and all of them failed to assess the false positive outcomes regarding significant associations due to the evaluation of multiple comparisons. Furthermore, these prior meta-analyses did not employ methods such as Bayesian false discovery probability (BFDP) and 95% predictive interval (PI) to assess the robustness of significant findings, leaving the possibility that some reported associations may represent false positives. Consequently, an updated meta-analysis was performed to systematically evaluate the association between TNF-α-308G/A and TNF-α-238G/A polymorphisms and the susceptibility of SLE.

## 2 Materials and methods

This study was not registered and a protocol was not prepared. However, the current meta-analysis was conducted according to the Preferred Reporting Items for Systematic Reviews and Meta-Analyses (PRISMA) guidelines (16).

### 2.1 Search strategy

We systematically searched PubMed, Embase, China National Knowledge Infrastructure (CNKI), and WanFang from database inception to up to October 16, 2025.

Moreover, we conducted the last search update on May 28, 2026, and no new studies were found. Search terms included “polymorphism,” “systemic lupus erythematosus,” and “Tumor necrosis factor,” as well as the corresponding MeSH (Medical Subjects Heading) terms. No restrictions were imposed on publication year or language. The full search strategy is available in Supplementary Table 1. Moreover, references of published systematic reviews and meta-analyses studies were also carefully verified.

### 2.2 Inclusion and exclusion criteria

The eligible studies must satisfy the following criteria: (1) case–control or cohort studies, (2) explored the association between the TNF-α-308G/A and TNF-α-238G/A polymorphisms and SLE susceptibility, and (3) provided the genotype data or the odds ratios (OR) with corresponding 95% confidence intervals (CI). Exclusion criteria as following: review articles, case reports, editorials, meta-analyses and systematic reviews, other SNPs, other diseases, animal studies, and duplicate data.

### 2.3 Data extraction and assessment of risk of bias

Two authors independently extracted and verified data from the original studies. Disagreement was reiterated and examined through the engagement of relevant experts. This study primarily extracted the following data: first author surname, year of publication, country, geographic region, ethnicity, sample size, source of controls, type of controls, matching, and genotypes distribution.

The methodological quality of the included studies was evaluated using Q-Genie tool (17). Each study was classified as poor, moderate, and good. Scores ≤35 indicate poor quality studies, >35 and ≤45 indicate studies of moderate quality, and >45 indicate good quality studies. The quality of the studies was independently evaluated by two investigators.

### 2.4 Statistical analysis

Pooled crude odds ratios (ORs) and 95% confidence intervals (CIs) were computed to assess the association on the TNF-α-308G/A and TNF-α-238G/A polymorphisms with the susceptibility of SLE. The pooled ORs were calculated applying the following five genetic models: (1) a dominant model ([GA + AA] vs. GG), (2) a recessive model (AA vs. [GG + GA]), (3) a heterozygous model (GA vs. GG), (4) a homozygous model (AA vs. GG), and (5) an allele model (A vs. G). Heterogeneity among studies was evaluated by calculating Q-statistic and *I*^2^ value (significant heterogeneity was regarded if *p* < 0.01 and/or *I*^2^ > 50%) (18). For each genetic model contrast, The ORs and 95% CIs were pooled applying random-effects model (19). This was deemed more appropriate than the fixed-effects model because the studies included in this meta-analysis represented samples from different populations. Moreover, the random-effects model is a more conservative choice when heterogeneity is present, whereas it reduces to the fixed effect model when heterogeneity is absent (20). We also reported the 95% Pls for the summary random effects estimates. Subgroup analyses were conducted according to ethnicity if heterogeneity among studies was significant. A sensitivity analysis was conducted by sequentially excluding each study and recalculating the pooled ORs and 95% CIs for all genetic models in order to evaluate whether any single study disproportionately influenced the pooled effect estimates. Moreover, we applied a data set [excluding studies of poor and moderate quality studies, Hardy–Weinberg disequilibrium (HWD) studies, and studies with a sample size of less than 100] to conduct the further sensitivity analysis. Hardy–Weinberg equilibrium (HWE) (*p* < 0.05) was considered as HWD was calculated according to chi-square goodness-of-fit test. Furthermore, we applied Begg’s funnel plot (21) and Egger’s regression asymmetry test (22) to identify publication bias. Then, a non-parametric “trim and fill” method (23) was applied if publication bias existed. Moreover, we employed a meta-regression analysis to explore the sources of heterogeneity among studies. All statistical analyses were calculated by using Stata 18.0 software (STATA Corporation, College Station, TX, United States) in this study.

### 2.5 Credibility of genetic association

To address the issue of multiple comparisons and reduce the likelihood of false-positive findings, we applied the Bayesian false discovery probability (BFDP) method (24). BFDP is a Bayesian analogue of the false discovery rate that calculates the posterior probability that a statistically significant association is false, given the observed data and a specified prior probability of association. In this study, a prior probability of 0.01, 0.001, and 0.00001 was assigned to the null hypothesis (i.e., no true association), reflecting the conservative assumption that genuine genetic associations are rare in complex diseases. A significant association was considered “positive” (i.e., noteworthy) if it met the following criteria: (1) statistically significant association were found in at least two genetic models, (2) all three BFDP values were <0.80, (3) the significant association was based on evidence from more than 1,000 cases, (4) *I*^2^ value was less than <75%, and (5) 95% PI excluded the null value. All other significant associations were considered as less-credible positives (25, 26).

## 3 Results

### 3.1 Characteristics of studies

**Figure 1:**
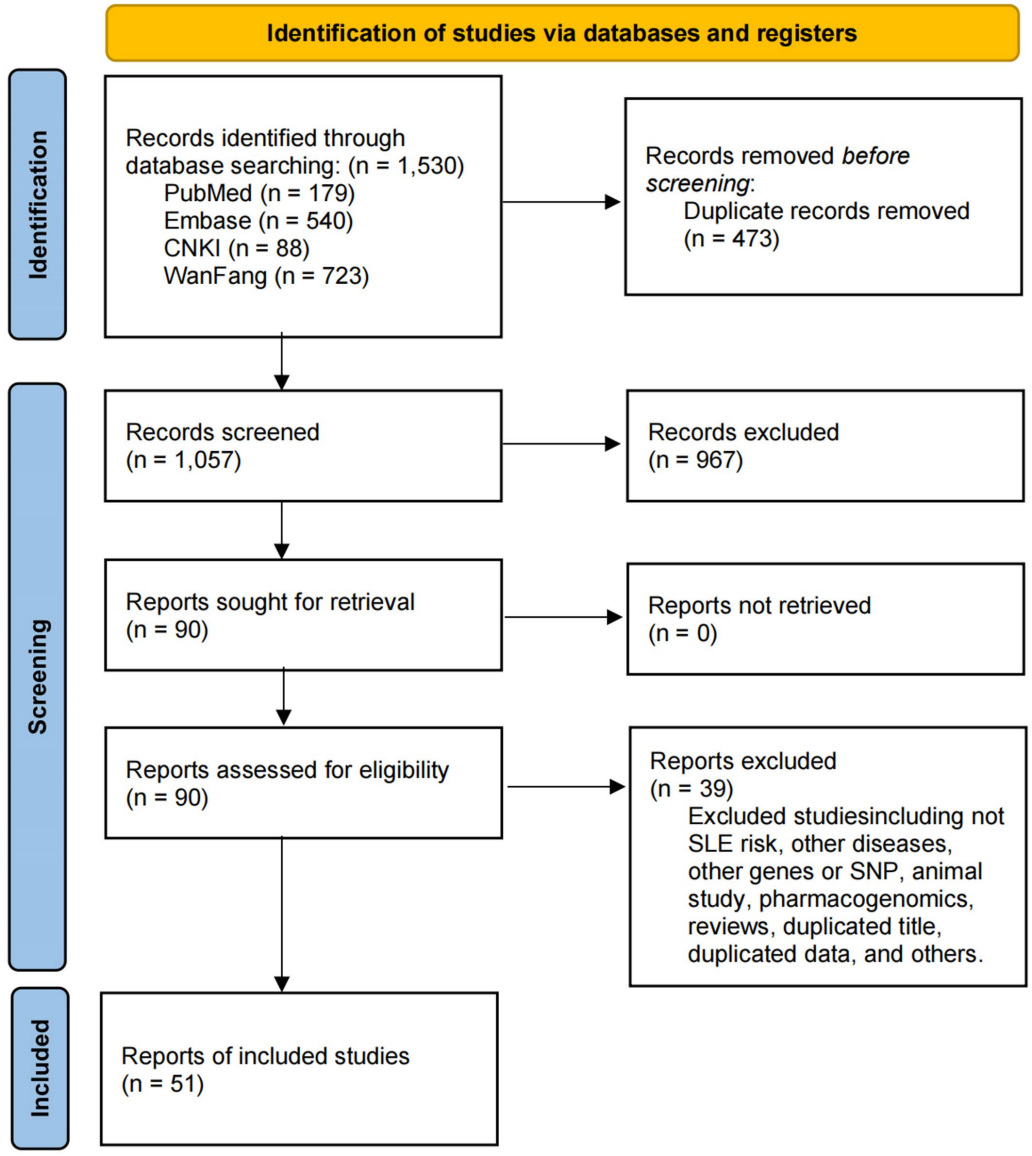
Flow diagram for identifying and including studies in this meta-analysis. * Consider, if feasible to do so, reporting the number of records identified from each database or register searched (rather than the total number across all databases/registers). ** If automation tools were used, indicate how many records were excluded by a human and how many were excluded by automation tools. Source: Page MJ, et al. BMJ 2021;372:n71. doi: 10.1136/bmj.n71. This work is licensed under CC BY 4.0. To view a copy of this license, visit https://creativecommons.org/licenses/by/4.0/

Figure 1 illustrates a flow diagram for the identification and inclusion of studies. This study incorporated 51 published original articles comprising 53 case–control studies, because one study (27) was repeated with other study (28). Furthermore, the current and published meta-analyses encompassing studies are explicitly detailed in Supplementary Table 2, while the principal characteristics of the included studies are enumerated in Supplementary Tables 3, 4. Fifty-one articles (11, 28–77) included 53 case–control studies to investigate the association between the TNF-α-308G/A polymorphism and the susceptibility of SLE (6,601 SLE cases and 8,948 controls; among which 3 studies were for Africans, 15 for Asians, 23 for Caucasians, 3 for Indians, and 9 for mixed populations); and 16 published articles (36, 37, 42, 44, 49, 54, 55, 57, 59, 61, 65, 70, 71, 73–75) involving 18 case–control studies to assess the association between the TNF-α-238G/A polymorphism and the susceptibility of SLE (2,495 SLE cases and 3,509 controls; among which 2 studies were for Africans, 1 for Asians, 7 for Caucasians, 2 for Indians, and 6 for mixed populations). Moreover, HWD of controls was observed in four studies (63, 69, 74, 76) for the TNF-α-308G/A polymorphism and four studies (44, 54, 65, 74) for the TNF-α-238G/A polymorphism. Furthermore, 41 studies were classified as having good quality for the TNF-α-308G/A polymorphism (Supplementary Tables 3, 5) and 15 studies were classified as having good quality for the TNF-α-238G/A polymorphism (Supplementary Tables 4, 5).

### 3.2 Quantitative synthesis

**Figure 2:**
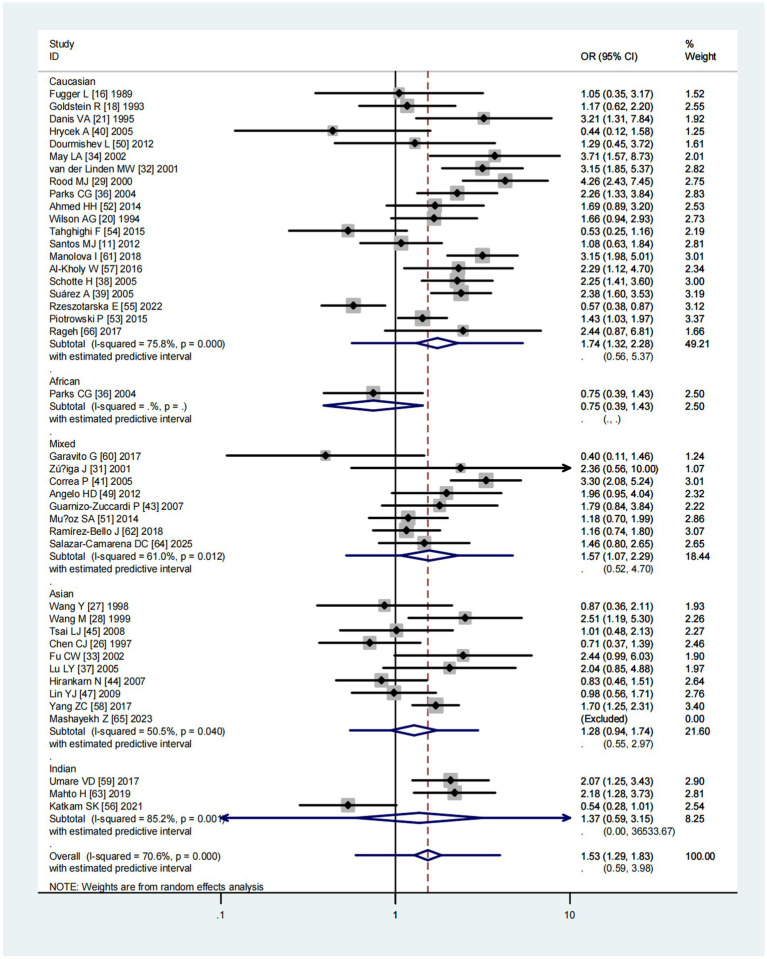
Forest plots of the TNF-α-308G/A polymorphism and risk of SLE in overall analysis and subgroup analysis by ethnicity (heterozygous model).

**Figure 3:**
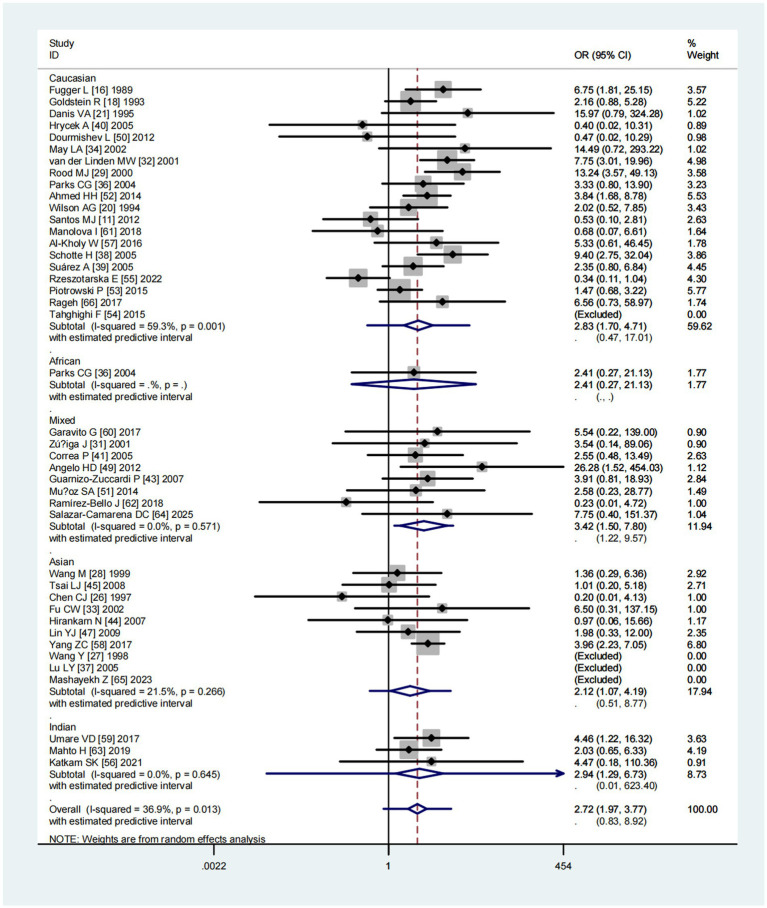
Forest plots of the TNF-α-308G/A polymorphism and risk of SLE in overall analysis and subgroup analysis by ethnicity (homozygous model).

**Figure 4:**
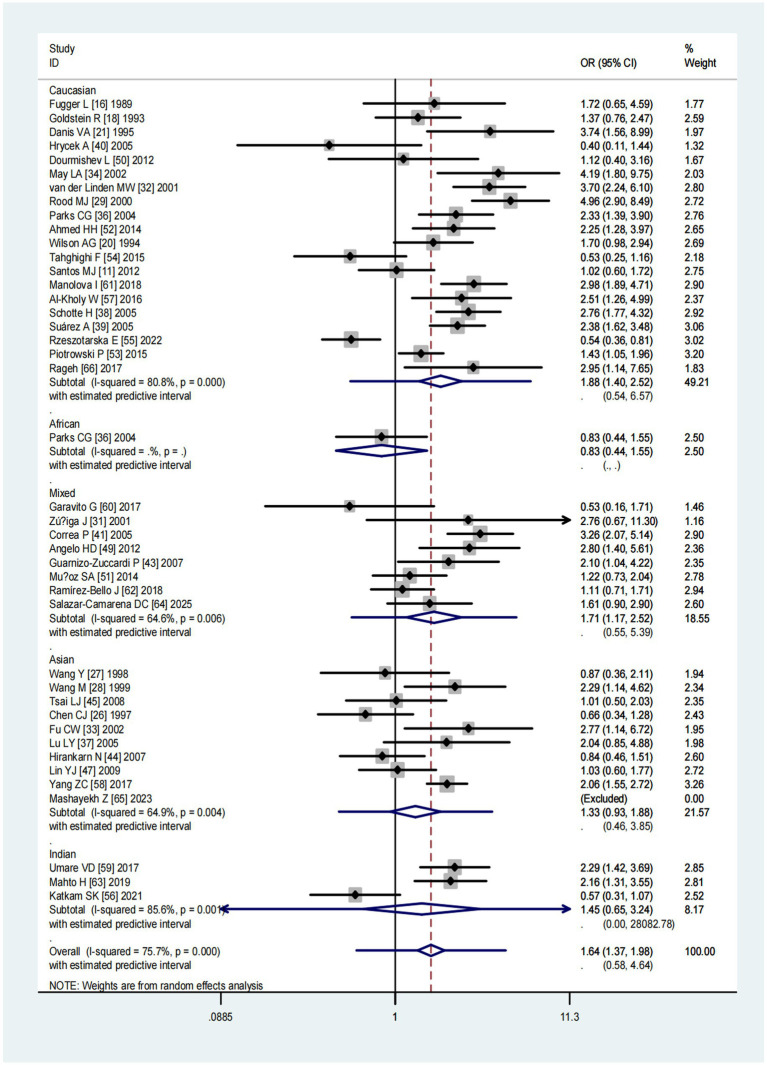
Forest plots of the TNF-α-308G/A polymorphism and risk of SLE in overall analysis and subgroup analysis by ethnicity (dominant model).

**Figure 5:**
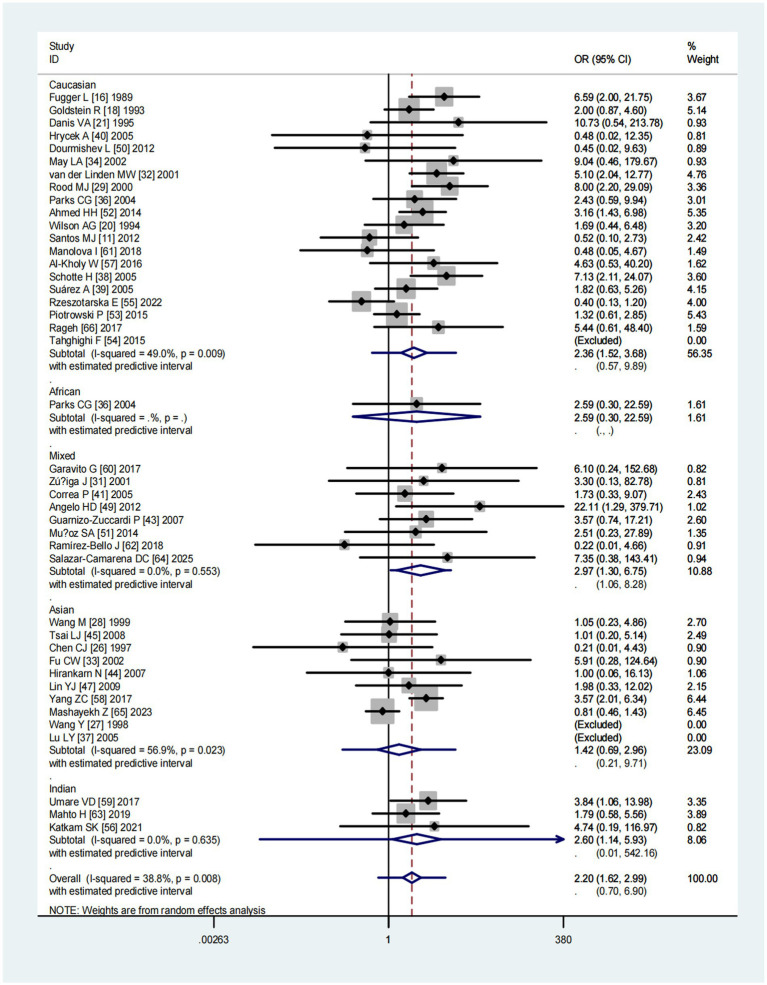
Forest plots of the TNF-α-308G/A polymorphism and risk of SLE in overall analysis and subgroup analysis by ethnicity (recessive model).

**Figure 6:**
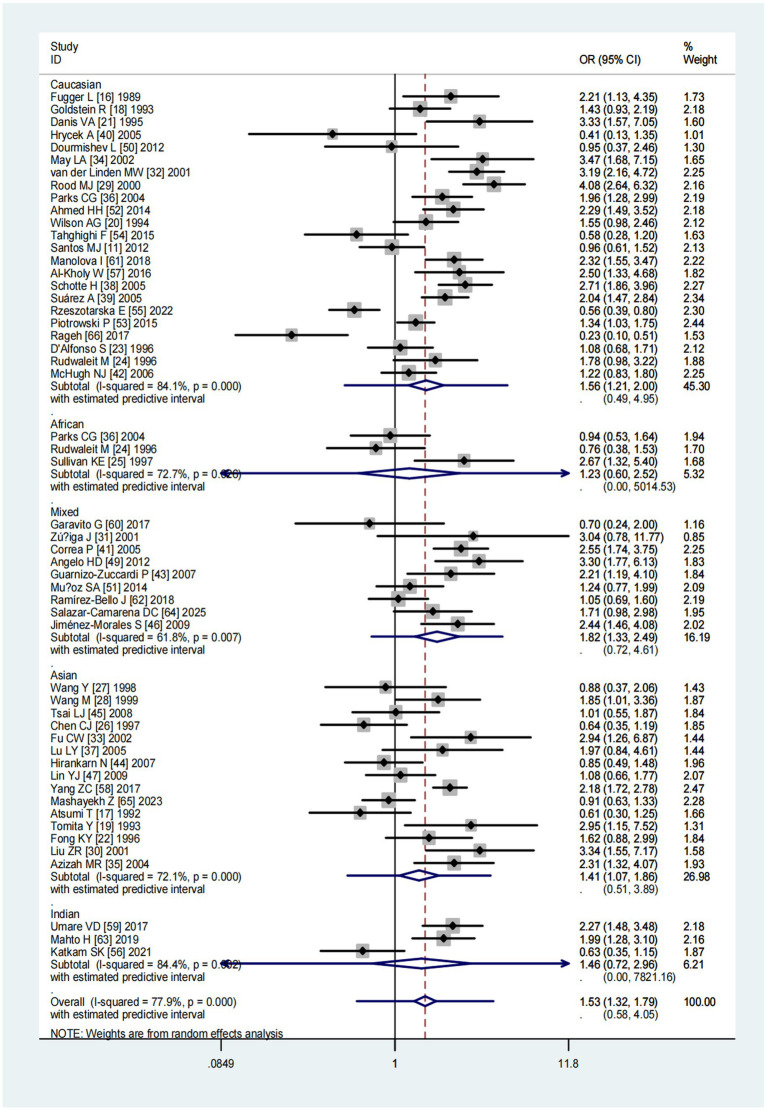
Forest plots of the TNF-α-308G/A polymorphism and risk of SLE in overall analysis and subgroup analysis by ethnicity (allele model).

Overall, our analysis revealed that the TNF-α-308G/A polymorphism had a significantly increased SLE susceptibility (heterozygous model: OR = 1.54, 95% CI: 1.29–1.83; homozygous model: OR = 2.72, 95% CI: 1.97–3.77; dominant model: OR = 1.64, 95% CI: 1.37–1.98; recessive model: OR = 2.20, 95% CI: 1.62–2.99; allele model: OR = 1.53, 95% CI: 1.32–1.79, Table 1; Figures 2–6) in overall population. Then, a significantly increased SLE susceptibility was also observed in Asians (homozygous model: OR = 2.12, 95% CI: 1.07–4.19; allele model: OR = 1.41, 95% CI: 1.06–1.86, Table 1; Figures 2–6), Caucasians (heterozygous model: OR = 1.74, 95% CI: 1.32–2.28; homozygous model: OR = 2.83, 95% CI: 1.70–4.71; dominant model: OR = 1.88, 95% CI: 1.40–2.52; recessive model: OR = 2.36, 95% CI: 1.52–3.68; allele model: OR = 1.56, 95% CI: 1.21–2.00, Table 1; Figures 2–6), Indians (homozygous model: OR = 2.94, 95% CI: 1.29–6.73; recessive model: OR = 2.60, 95% CI: 1.14–5.93, Table 1; Figures 2–6), and mixed populations (heterozygous model: OR = 1.57, 95% CI: 1.07–2.29; homozygous model: OR = 3.42, 95% CI: 1.50–7.80; dominant model: OR = 1.71, 95% CI: 1.17–2.52; recessive model: OR = 2.97, 95% CI: 1.31–6.75; allele model: OR = 1.82, 95% CI: 1.33–2.49, Table 1; Figures 2–6). Subsequently, we performed a sensitivity analysis and determined that the results remained unchanged when each study was excluded individually (data not shown). Moreover, when we excluded studies of high-risk bias, HWD studies, and studies with a sample size of less than 100, the results remained consistent (Table 1).

**Table 1:** Pooled results on the association between the TNF-α-308G/A (rs1800629) polymorphism and SLE risk.

Variable	*n* (cases/controls)	Heterozygous model		Homozygous model		Dominant model		Recessive model		Allele model	
		OR (95% CI) *P*_h_/*I*^2^ (%)		OR (95% CI) *P*_h_/*I*^2^ (%)		OR (95% CI) *P*_h_/*I*^2^ (%)		OR (95% CI) *P*_h_/*I*^2^ (%)		OR (95% CI) *P*_h_/*I*^2^ (%)	
Overall	53 (6,601/8948)	**1.54 (1.29, 1.83)**	<0.001/70.6	**2.72 (1.97, 3.77)**	0.013/36.9	**1.64 (1.37, 1.98)**	<0.001/75.7	**2.20 (1.62, 2.99)**	0.008/38.8	**1.53 (1.32, 1.79)**	<0.001/77.9
Ethnicity											
African	3 (257/242)	0.75 (0.39, 1.43)	–	2.41 (0.27, 21.13)	–	0.83 (0.44, 1.55)	–	2.59 (0.30, 22.59)	–	1.23 (0.60, 2.52)	0.026/72.7
Asian	15 (1855/1988)	1.28 (0.94, 1.74)	0.040/50.5	**2.12 (1.07, 4.19)**	0.266/21.5	1.33 (0.93, 1.88)	0.004/64.9	1.42 (0.69, 2.96)	0.023/56.9	**1.41 (1.07, 1.86)**	<0.001/72.1
Caucasian	23 (2,388/3916)	**1.74 (1.32, 2.28)**	<0.001/75.8	**2.83 (1.70, 4.71)**	0.001/59.3	**1.88 (1.40, 2.52)**	<0.001/80.8	**2.36 (1.52, 3.68)**	0.009/49.0	**1.56 (1.21, 2.00)**	<0.001/84.1
Indian	3 (606/743)	1.37 (0.59, 3.15)	<0.001/85.2	**2.94 (1.29, 6.73)**	0.645/0.0	1.45 (0.65, 3.24)	0.001/85.6	**2.60 (1.14, 5.93)**	0.635/0.0	1.46 (0.72, 2.96)	0.002/84.4
Mixed	9 (1,495/2059)	**1.57 (1.07, 2.29)**	0.012/61.0	**3.42 (1.50, 7.80)**	0.571/0.0	**1.71 (1.17, 2.52)**	0.006/64.6	**2.97 (1.31, 6.75)**	0.553/0.0	**1.82 (1.33, 2.49)**	0.007/61.8
Sensitivity analysis											
Overall	34 (4,490/6225)	**1.55 (1.22, 1.96)**	<0.001/76.6	**2.68 (1.68, 4.28)**	0.004/47.9	**1.65 (1.29, 2.12)**	<0.001/80.3	**2.32 (1.52, 3.54)**	0.028/37.9	**1.50 (1.23, 1.84)**	<0.001/80.9
Ethnicity											
African	3 (257/242)	0.75 (0.39, 1.43)	–	2.41 (0.27, 21.13)	–	0.83 (0.44, 1.55)	–	2.59 (0.30, 22.59)	–	1.23 (0.60, 2.52)	0.026/72.7
Asian	8 (902/958)	1.25 (0.85, 1.82)	0.062/50.0	1.29 (0.56, 2.96)	0.716/0.0	1.26 (0.85, 1.85)	0.040/54.5	1.20 (0.52, 2.74)	0.747/0.0	1.27 (0.92, 1.74)	0.044/51.4
Caucasian	14 (1,615/2735)	**1.94 (1.32, 2.86)**	<0.001/81.9	**2.93 (1.36, 6.30)**	<0.001/71.6	**2.11 (1.39, 3.20)**	<0.001/85.9	**2.40 (1.23, 4.71)**	0.002/64.4	**1.55 (1.10, 2.18)**	<0.001/88.1
Indian	2 (402/519)	1.07 (0.28, 4.02)	0.001/90.6	**4.46 (1.34, 14.85)**	0.999/0.0	1.16 (0.30, 4.51)	0.001/91.6	**3.96 (1.19, 13.11)**	0.905/0.0	1.22 (0.35, 4.27)	0.001/91.5
Mixed	7 (1,314/1781)	**1.59 (1.00, 2.54)**	0.007/68.9	**3.58 (1.32, 9.71)**	0.339/11.9	**1.76 (1.10, 2.81)**	0.004/71.2	**3.07 (1.11, 8.50)**	0.319/14.9	**1.89 (1.32, 2.69)**	0.001/91.5

SLE, Systemic lupus erythematosus; OR, Odds ratio; CI, Confidence interval; Ph, *P*-value for heterogeneity; I^2^, Inconsistency index. Bold values in both the main analysis and sensitivity analysis indicate statistically significant associations (*P* < 0.05, 95% confidence interval does not include 1). Significant results in the sensitivity analysis further confirm the robustness of the main findings.

Then, our analysis found that the TNF-α-238G/A polymorphism was not associated with SLE susceptibility in overall analysis (heterozygous model: OR = 1.15, 95% CI: 0.63–2.08; homozygous model: OR = 1.56, 95% CI: 0.66–3.67; dominant model: OR = 1.16, 95% CI: 0.68–1.98; recessive model: OR = 1.52, 95% CI: 0.64–3.58; allele model: OR = 1.09, 95% CI: 0.79–1.58; Table 2). Moreover, we also did not observe a significant association in subgroup analysis according to ethnicity (Table 2). However, when we conducted a sensitivity analysis according to dataset, we found that the TNF-α-238G/A polymorphism had a significantly increased SLE susceptibility in overall analysis (heterozygous model: OR = 1.54, 95% CI: 1.04–2.28; dominant model: OR = 1.52, 95% CI: 1.06–2.17; Table 2) and mixed populations (heterozygous model: OR = 1.93, 95% CI: 1.37–2.72; dominant model: OR = 1.93, 95% CI: 1.38–2.70, allele model: OR = 1.49, 95% CI: 1.08–2.04; Table 2).

**Table 2:** Pooled results on the association between the TNF-α-238G/A (rs361525) polymorphism and SLE risk.

Variable	*n* (cases/controls)	Heterozygous model		Homozygous model		Dominant model		Recessive model		Allele model	
		OR (95% CI) *P*_h_/*I*^2^ (%)		OR (95% CI) *P*_h_/*I*^2^ (%)		OR (95% CI) *P*_h_/*I*^2^ (%)		OR (95% CI) *P*_h_/*I*^2^ (%)		OR (95% CI) *P*_h_/*I*^2^ (%)	
Overall	18 (2,495/3509)	1.15 (0.63, 2.08)	<0.001/81.3	1.56 (0.66, 3.67)	0.951/0.0	1.0.16 (0.68, 1.98)	<0.001/79.6	1.52 (0.64, 3.58)	0.948/0.0	1.09 (0.75, 1.58)	<0.001/73.3
Ethnicity											
Caucasian	7 (597/1119)	0.45 (0.08, 2.43)	0.011/77.9	–	–	0.59 (0.17, 1.97)	0.006/76.1	–	–	0.58 (0.29, 1.18)	0.010/67.0
Mixed	6 (1,147/1657)	1.25 (0.47, 3.32)	<0.001/83.9	1.67 (0.52, 5.39)	0.874/0.0	1.24 (0.48, 3.23)	<0.001/84.0	1.58 (0.49, 5.10)	0.897/0.0	1.23 (0.65, 2.33)	<0.001/78.6
Sensitivity analysis											
Overall	13 (2057/2600)	**1.54 (1.04, 2.28)**	0.100/43.6	1.92 (0.52, 6.60)	0.725/0.0	**1.52 (1.06, 2.17)**	0.116/39.5	1.79 (0.52, 6.14)	0.731/0.0	1.24 (0.92, 1.66)	0.027/49.1
Ethnicity											
Caucasian	5 (514/919)	1.22 (0.12, 12.45)	–	–	–	0.77 (0.38, 1.57)	0.275/16.1	–	–	0.71 (0.40, 1.26)	0.143/44.8
Mixed	4 (996/1172)	**1.93 (1.37, 2.72)**	0.466/0.0	1.53 (0.35, 6.65)	0.604/0.0	**1.93 (1.38, 2.70)**	0.443/0.0	1.43 (0.33, 6.22)	0.607/0.0	**1.49 (1.08, 2.04)**	0.278/22.1

SLE, Systemic lupus erythematosus; OR, Odds ratio; CI, Confidence interval; Ph, *P*-value for heterogeneity; I^2^, Inconsistency index. Bold values in both the main analysis and sensitivity analysis indicate statistically significant associations (*P* < 0.05, 95% confidence interval does not include 1). Significant results in the sensitivity analysis further confirm the robustness of the main finding.

### 3.3 Heterogeneity analysis and publication bias

**Figure 7:**
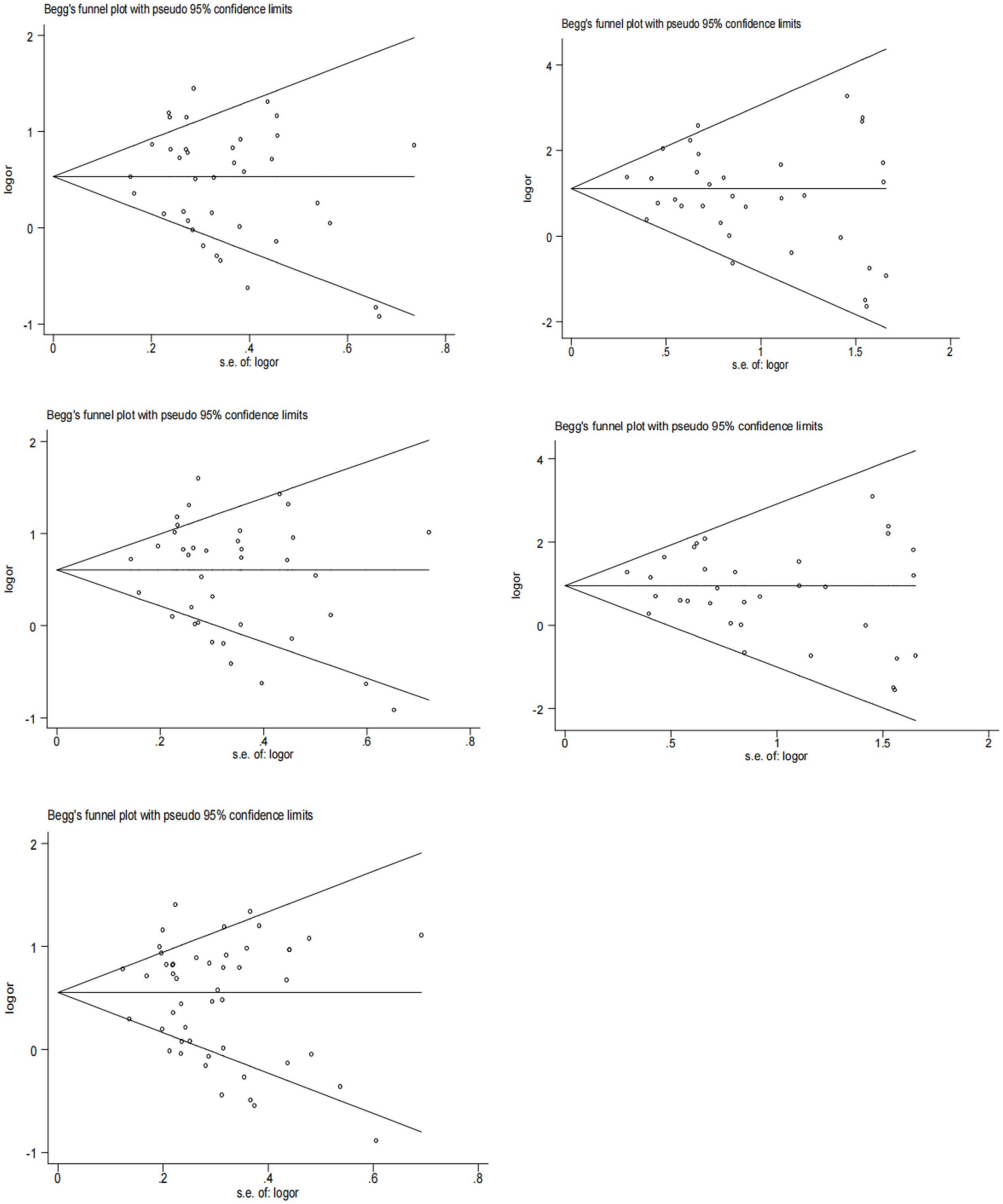
The Begg’s funnel plot on the TNF-α-308G/A polymorphism with SLE risk in overall analysis (heterozygous model; homozygous model; dominant model; recessive model; allele model).

**Figure 8:**
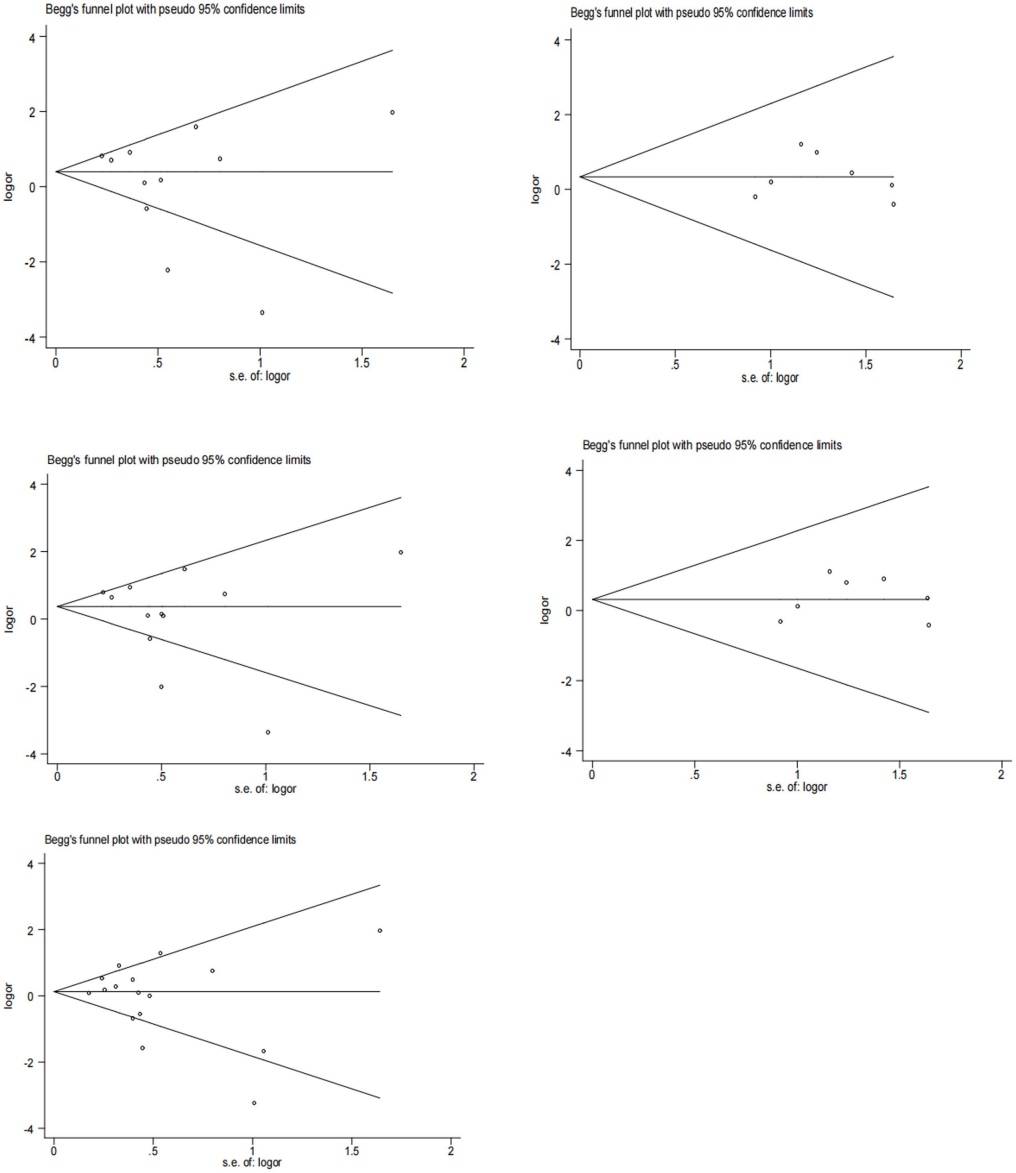
The Begg’s funnel plot on the TNF-α-238G/A polymorphism with SLE risk in overall analysis (heterozygous model; homozygous model; dominant model; recessive model; allele model).

Significant heterogeneity among original studies was observed (Tables 1, 2). We found that type of controls (heterozygous model: *p* = 0.049 for the TNF-α − 308G/A polymorphism; dominant model: *p* = 0.047 for the TNF-α-308G/A polymorphism; allele model: *p* = 0.019 for the TNF-α-238G/A) was source of heterogeneity for the according to the results of meta-regression analysis. No significant publication bias was observed according to the Begg’s funnel plot (Figures 7, 8) and Egger’s test (*p* > 0.05 in all genetic models) in overall analysis.

### 3.4 Credibility of the identified genetic associations

We found that the 95% PI excluded the null value for only four of these significant association, as shown in Table 3. However, BFDP values of two of these significant association were more than 0.80 (Table 3). After confidence evaluation, all significant associations were considered as less-credible positives according to above criteria (Table 3).

**Table 3:** Credibility of the current meta-analysis.

Variables	Genetic model	OR (95% CI)	Credibility				95% PI
			*I*^2^ (%)	Prior probability of BFDP value			
				0.01	0.001	0.00001	
TNF-α-308G/A (rs1800629)							
Overall	Heterozygous model	1.54 (1.29, 1.83)	70.6	0.005	0.049	0.837	0.59, 3.98
	Homozygous model	2.72 (1.97, 3.77)	36.9	<0.001	<0.001	0.022	0.83, 8.92
	Dominant model	1.64 (1.37, 1.98)	75.7	0.002	0.015	0.605	0.58, 4.64
	Recessive model	2.20 (1.62, 2.99)	38.8	0.003	0.028	0.743	0.70, 6.90
	Allele model	1.53 (1.32, 1.79)	77.9	0.001	0.007	0.413	0.58, 4.05
Ethnicity							
Asian	Dominant model	2.12 (1.07, 4.19)	21.5	0.972	0.997	1.000	0.51, 8.77
	Allele model	1.41 (1.07, 1.86)	72.1	0.962	0.996	1.000	0.51, 3.89
Caucasian	Heterozygous model	1.74 (1.32, 2.28)	75.8	0.171	0.675	0.995	0.56, 5.37
	Homozygous model	2.83 (1.70, 4.71)	59.3	0.244	0.765	0.997	0.47, 17.01
	Dominant model	1.88 (1.40, 2.52)	80.8	0.085	0.484	0.989	0.54, 6.57
	Recessive model	2.36 (1.52, 3.68)	49.0	0.356	0.848	0.998	0.57, 9.89
	Allele model	1.56 (1.21, 2.00)	84.1	0.563	0.929	0.999	0.49, 4.95
Indian	Homozygous model	2.94 (1.29, 6.73)	0.0	0.954	0.995	1.000	0.01, 623.40
	Recessive model	2.60 (1.14, 5.93)	0.0	0.969	0.997	1.000	0.01, 542.16
Mixed	Heterozygous model	1.57 (1.07, 2.29)	61.0	0.963	0.996	1.000	0.52, 4.70
	Homozygous model	3.42 (1.50, 7.80)	0.0	0.915	0.991	1.000	**1.22, 9.57**
	Dominant model	1.71 (1.17, 2.52)	64.6	0.919	0.991	1.000	0.55, 5.39
	Recessive model	2.97 (1.31, 6.75)	0.0	0.950	0.995	1.000	**1.06, 8.28**
	Allele model	1.82 (1.33, 2.49)	61.8	0.356	0.848	0.998	0.72, 4.61
Sensitivity analysis							
Overall	Heterozygous model	1.55 (1.22, 1.96)	76.6	0.439	0.888	0.999	0.50, 4.83
	Homozygous model	2.68 (1.68, 4.28)	47.9	0.157	0.653	0.995	0.48, 14.95
	Dominant model	1.65 (1.29, 2.12)	80.3	0.233	0.754	0.997	0.49, 5.63
	Recessive model	2.32 (1.52, 3.54)	37.9	0.266	0.785	0.997	0.58, 9.35
	Allele model	1.50 (1.23, 1.84)	80.9	0.265	0.785	0.997	0.51, 4.47
Ethnicity							
Caucasian	Heterozygous model	1.94 (1.32, 2.86)	81.9	0.672	0.954	1.000	0.49, 7.69
	Homozygous model	2.93 (1.36, 6.30)	71.6	0.931	0.993	1.000	0.22, 38.65
	Dominant model	2.11 (1.39, 3.20)	85.8	0.558	0.927	0.999	0.46, 9.73
	Recessive model	2.40 (1.23, 4.71)	64.4	0.947	0.995	1.000	0.28, 20.79
	Allele model	1.55 (1.10, 2.18)	88.1	0.949	0.995	1.000	0.39, 6.12
Indian	Homozygous model	4.46 (1.34, 14.85)	0.0	0.972	0.997	1.000	– *
	Recessive model	3.96 (1.19, 13.11)	0.0	0.977	0.998	1.000	– *
Mixed	Heterozygous model	1.59 (1.00, 2.54)	68.9	0.981	0.998	1.000	0.37, 6.87
	Homozygous model	3.58 (1.32, 9.71)	11.9	0.963	0.996	1.000	0.56, 23.05
	Dominant model	1.76 (1.10, 2.81)	71.2	0.959	0.996	1.000	0.40, 7.79
	Recessive model	3.07 (1.11, 8.50)	14.9	0.977	0.998	1.000	0.42, 22.53
	Allele model	1.89 (1.32, 2.69)	65.9	0.529	0.998	0.999	0.64, 5.56
TNF-α-238G/A (rs361525) 0.919							
Sensitivity analysis							
Overall	Heterozygous model	1.54 (1.04, 2.28)	43.6	0.974	0.997	1.000	0.56, 4.22
	Dominant model	1.52 (1.06, 2.17)	39.5	0.967	0.997	1.000	0.63, 3.68
Mixed	Heterozygous model	1.93 (1.37, 2.72)	0.0	0.349	0.844	0.998	0.21, 17.75
	Dominant model	1.93 (1.38, 2.70)	0.0	0.286	0.802	0.998	0.22, 17.01
	Allele model	1.49 (1.08, 2.04)	22.1	0.954	0.995	1.000	0.57, 3.88

OR, Odds ratio; CI, Confidence interval; I², Inconsistency index; BFDP, Bayesian false discovery probability; PI, Predictive interval. BFDP was calculated with prior probabilities of 0.01, 0.001, and 0.00001. Credible positives met: significance in ≥2 models, all BFDP <0.80, >1000 cases, I² <75%, and 95% PI excluding null. *Inestimable predictive distribution with <3 studies.

## 4 Discussion

Fugger et al. (29) in 1989 first reported the association on the TNF-α-308G/A polymorphism with SLE susceptibility in Caucasians. Several years later, D’Alfonso et al. (36) in 1996 first investigate the correlation between TNF-α-238G/A polymorphism and SLE susceptibility in Caucasians. Since then, many original studies examined the TNF-α-308G/A and TNF-α-238G/A polymorphisms with SLE susceptibility in different ethnicities. Unfortunately, the results of these published original studies were inconsistent. Although seven previously published meta-analyses assessed the association between the TNF-α-308G/A and TNF-α-238G/A polymorphisms with SLE susceptibility. However, results of these meta-analyses also yielded a significant disagreement. Therefore, we performed an updated meta-analysis to further investigate the association on the TNF-α-308G/A and TNF-α-238G/A polymorphisms with SLE susceptibility.

Overall, the TNF-α-308G/A polymorphism was associated with increased SLE susceptibility in overall analysis, Asians, Caucasians, Indians, and mixed populations. No significant association was found for the TNF-α-238G/A polymorphism with SLE susceptibility. Significant heterogeneity was found in this study. The results of meta-regression analysis showed that type of controls was source of heterogeneity for the TNF-α-308G/A polymorphism only in two genetic models and the TNF-α-238G/A polymorphism only in one genetic model. In this study, HWD, high risk of bias, and small sample studies were not source of heterogeneity according to the results of meta-regression analysis. However, HWD may be genotyping errors and selection bias in molecular epidemiological studies. Small sample and high risk of bias studies were easier to accept if there were positive reports as they tend to yield false-positive results because they may be not rigorous and are often of low-quality. Therefore, we applied a data set [excluding studies of high-risk bias, Hardy–Weinberg disequilibrium (HWD) studies, and studies with a sample size of less than 100] to conduct the further sensitivity analysis. In the further sensitivity analysis, we observed that the TNF-α-238G/A polymorphism was associated with increased SLE susceptibility in overall analysis and mixed populations. This was an attempt to avoid random errors and confounding bias that sometimes distorted the results of molecular epidemiological studies. However, this meta-analysis was conducted by using several different genetic models at the expense of multiple comparisons, under these circumstances, the pooled *p*-value must be adjusted (78). Wakefield et al. (24) in 2007 offered a precise Bayesian measure of false discovery on the genetic epidemiology studies. Therefore, BFDP value was applied to evaluate the false positive associations in this study. We also reported the 95% Pls for the summary random effects estimates, which further account for heterogeneity between studies and indicate the uncertainty for the effect that would be expected in a new study examining that same association (79). The 95% PI shows where the true effects are for 95% of the studies from the population of studies in the future. Unfortunately, the 95% PI excluded the null value for only two of these significant association. However, BFDP values of four of these significant association were more than 0.80. Therefore, all significant associations were considered as less-credible positives in this study.

Six published meta-analyses (69, 80–84) reported the association between the TNF-α-308G/A polymorphism and SLE susceptibility. Of these, Sekar et al. (83) in 2025 conducted an association of 31 publications including 1,647 SLE cases and 1772 controls, and showed that the TNF-α-308 G/A polymorphism was associated with increased SLE susceptibility in overall population, particularly in Asians and Caucasians; Chen et al. (80) in 2019 identified 26 studies of 3,051 SLE cases and 4,232 controls in all populations, and demonstrated that the TNF-α-308 G/A polymorphism had a significantly increased SLE susceptibility in European and Latin American populations; Yang et al. (69) in 2017 selected 41 studies (involving 4,799 SLE cases and 6,635 controls) to evaluate the TNF-α-308 G/A polymorphism with SLE susceptibility and they observed that the TNF-α-308 G/A polymorphism was associated with increased SLE susceptibility in European, Asian, South American, and North American populations; Pan et al. (81) in 2012 found that the TNF-α-308G/A polymorphism was associated with a significantly increased SLE susceptibility in European, South American, and Mexican populations; Zou et al. (82) in 2011 observed that the TNF-α-308G/A polymorphism was associated with increased SLE susceptibility in Asians, especially in Chinese population; Lee et al. (85) in 2006 only obtained a significant association in European populations. Moreover, two published meta-analyses (80, 86) reported the association on the TNF-α-238G/A polymorphism with SLE susceptibility. One study (80) indicated that the TNF-α-238G/A polymorphism had a significantly increased SLE susceptibility in Latin Americans and other study (86) observed a contradictory result in different genetic models of Caucasians. These previous results were not consistent. An obvious inconsistency was found in classification of races among these previously published meta-analyses (Supplementary Table 2). Moreover, we still found an obvious difference on the HWE in control population (Supplementary Table 2). Furthermore, all previously published meta-analyses involved incomplete studies and did not explore false significant results on the multiple comparison. Therefore, results of previously published meta-analyses may be incredible, and an updated meta-analysis was performed using a larger sample size to further explore the association between the TNF-α-308G/A and TNF-α-238G/A polymorphisms with SLE susceptibility.

Despite all our efforts to improve our research, our study has limitations. First, the analysis was based solely on published literature, and the potential for publication bias due to unpublished negative results cannot be entirely ruled out. Second, for some subgroup analyses, such as those for the TNF-α-238G/A polymorphism or certain ethnic groups, the number of included studies or the sample size remains relatively limited, which may affect statistical power and the stability of the conclusions. Third, due to insufficient data in the original studies, we were unable to perform more in-depth subgroup analyses, nor could we conduct pooled analyses adjusted for potential confounders.

In summary, this study indicates that the TNF-α-308G/A and TNF-α-238G/A polymorphisms may be not associated with increased SLE susceptibility. The increased susceptibility of SLE is most likely due to false-positive results.

## Data Availability

The datasets presented in this study can be found in online repositories. The names of the repository/repositories and accession number(s) can be found in the article/Supplementary material.
